# The Role of Histone Methyltransferases and Long Non-coding RNAs in the Regulation of T Cell Fate Decisions

**DOI:** 10.3389/fimmu.2018.02955

**Published:** 2018-12-13

**Authors:** Joseph M. Gaballa, Manuel Bonfim Braga Neto, Guilherme Piovezani Ramos, Adebowale O. Bamidele, Michelle M. Gonzalez, Mary R. Sagstetter, Olga F. Sarmento, William A. Faubion

**Affiliations:** Division of Gastroenterology and Hepatology, Mayo Clinic, Rochester, MN, United States

**Keywords:** epigenetics, EZH2, G9a, long non-coding RNAs, PRC1, PRC2, T cell

## Abstract

T cell lineage decisions are critical for the development of proper immune responses to pathogens as well as important for the resolution of inflammatory responses. This differentiation process relies on a combination of intrinsic and extrinsic factors converging upon epigenetic regulation of transcriptional networks relevant to specific T cell lineages. As these biochemical modifications represent therapeutic opportunities in cancer biology and autoimmunity, implications of writers and readers of epigenetic marks to immune cell differentiation and function are highly relevant. Given the ready adoption of histone methyltransferase inhibitors in the clinic, we focus this review on the role of three histone modifying complexes: PRC-1, PRC-2, and G9A in modulating T cell fate decisions. Furthermore, we explore the role of long non-coding RNAs in regulating these processes, and discuss recent advances and challenges of implementing epigenetic therapies into clinical practice.

## Background

The immune system comprises a large number of cell types that have the ability to respond to external environmental cues and adopt a wide variety of cell fates. These lineage decisions are critical for the development of proper immune responses to pathogens as well as resolution of inflammatory responses. As part of the adaptive immune system, T cells have the capacity to respond to the external environment by modulating the expression of lineage specific factors which are critical for protecting against a wide variety of pathogens. For the development of distinct T cell lineages, naive CD4+ T cells must convert the extrinsic instructions provided by encounters with antigen-presenting cells into cell-intrinsic changes (1). These intrinsic changes are largely facilitated by transcription factors that directly induce or repress gene networks and drive T cell differentiation (2). Emerging data demonstrates that lineage specific transcription factors recruit epigenetic complexes to regulate gene expression over multiple rounds of cell division, and their roles are indispensable for maintaining T cell homeostasis.

Deregulation of epigenetic pathways is a feature of many cancers, autoimmune diseases, and neurodegenerative disorders (3–5). The reversible nature of epigenetic modifications makes them attractive targets for pharmacological intervention, and indeed drugs targeting histone-modifying complexes, such as Enhancer of Zeste Homolog 2 (EZH2), are currently being evaluated in patients for treatment of malignancy (6) and immune-mediated conditions (7, 8). While recent clinical trials have demonstrated a favorable safety profile of selective inhibition of EZH2 (6), a comprehensive understanding of the role that epigenetic modifying complexes play in the development and function of different immune cell types is relevant to the development and safety of epigenetic therapeutics. Here we review the role of three histone modifying complexes: PRC-1, PRC-2, and G9A in modulating T cell fate decisions. Furthermore, we explore the role of long non-coding RNAs in regulating these processes, and discuss recent advances and challenges associated with implementing epigenetic therapies in clinical practice.

## PRC1, PRC2, G9a, and Long Non-coding RNAs

### PRC1

The Polycomb-Group proteins, Polycomb Repressive Complex 1 (PRC1) and 2 (PRC2), mediate post-translational modifications (PTMs) of histones required for cell differentiation and development through the regulation of chromatin structure and gene expression. PRC1 is a multimeric protein complex containing the core proteins RING1A/B, and Polycomb-group ring finger (PCGF) proteins such as Bmi-1 (PCGF4) and Mel-18 (PCGF2). PRC1 functions to mono-ubiquitinate lysine 119 on histone H2A (H2AKub119), an epigenetic mark that is associated with transcriptional repression (9). Bmi-1 specifically is highly enriched in pericentric heterochromatin which is required for chromatin compaction and silencing (10). Although Ring1A/B is the catalytic subunit of PRC1, knockdown of Bmi-1 results in a significant loss of H2A ubiquitylation, demonstrating the important role that it plays in facilitating the enzymatic function of PRC1 (11). In the canonical or hierarchical model of Polycomb (PcG)-mediated transcription regulation, PRC1 is primarily described as the maintenance complex which silences target genes previously marked by the initiator complex, PRC2. More recently, a histone-independent role of Bmi-1 in driving NF-κB signaling has been reported (12). An interesting story is also evolving related to a PRC2-independent role for PRC1 in the maintenance of 3D genome structure through association with super-enhancers (13, 14). No immune cell specific data has yet emerged related to these exciting areas of investigation.

### PRC2

PRC2 modulates chromatin dynamics via the tri-methylation of lysine 27 on histone 3 (H3K27Me3), which is associated with transcriptional repression. EZH2, ubiquitously expressed by many mammalian cell-types, is the enzymatic subunit of PRC2 which contains other supporting non-catalytic proteins namely Suppressor of Zeste (SUZ12), embryonic ectoderm development (EED), Adipocyte Binding Protein 2 (AEBP2) and Retinoblastoma protein Associated protein 46 and 48 (RpAp46/48) (15). H3K27me3 recruits protein complexes involved in chromatin compaction and is associated with inactive genes (16). Histone-independent functions of PRC2 have also been reported to play important roles in regulating transcription factor stability and T cell receptor-mediated signaling (17–20). While EZH2 has a role in normal cellular and tissue function, studies involving EZH2 overexpression or genetic mutations show that EZH2 is critical in the development and progression of a variety of cancers (21–29). EZH2 is most frequently associated with the silencing of tumor suppressor genes, and decreased expression of PRC-target genes are associated with poor prognosis (30, 31). Thus, derepression of these genes using selective EZH2 enzymatic inhibitors or disruptors of PRC2 stability are likely to improve clinical outcomes, and are currently being explored in preclinical or clinical studies for cancer therapy (32–38).

### G9a

The histone methyltransferase G9a and the related G9a-like protein (GLP) form a heterodimeric complex to catalyze mono and di-methylation of lysine 9 on histone 3 (H3K9me1 & H3K9me2) at euchromatin *in vivo* (39). G9a and GLP are encoded by the *EHMT2* and *EHMT1* genes, respectively, both of which contain a SET domain necessary for the methylation of lysine residues. G9a has been shown to play a larger role in H3K9me2 methylation *in vivo*, but levels of H3K9me1 and H3K9me2 are severely reduced in both G9a and GLP knockout models (39). Furthermore, G9a has been shown to promote gene activation through a methyltransferase-independent fashion in different settings, including type II cytokine production in helper T cells, possibly by acting as a scaffold to recruit transcriptional machinery (40, 41). G9a/GLP-mediated H3K9me2 has been associated with cognition and adaptive behavior, germ cell development and meiosis, embryo development, cocaine-induced plasticity, tumor cell growth and metastasis, and more recently the immune response reviewed below (39, 42).

### Long Non-coding RNAs

Non-coding RNA's have emerged as an exciting new frontier of gene regulation in the immune system. It is now known that 75–90% of the human genome transcriptome is comprised of non-coding RNAs (43, 44). Long non-coding RNAs are defined as transcripts with minimal coding potential that are composed of more than 200 nucleotides; an arbitrary cutoff that distinguishes them from microRNAs (< 200 nucleotides). Over 15,000 lncRNA genes have been annotated, although only 159 lncRNAs have known function^1^,^2^ (45), highlighting a critical gap in knowledge in the field. They can be classified based on their position relative to protein coding genes as intergenic, intronic and antisense (46). Like mRNAs, long non-coding RNA's undergo transcription by RNA polymerase II, are 5′ capped, spliced and polyadenylated. However, distinct from mRNA, they lack canonical ORFs (and, therefore have minimal protein-coding potential), tend to be shorter in size, have lower expression levels, fewer exons and can localize to the nucleosome, chromatin or cytoplasm. For example, long intergenic non-coding RNAs localize primarily in the nucleus, in contrast to mRNAs which are primarily localized in the cytoplasm where they undergo translation (47). Furthermore, lncRNAs function by interacting with DNA, RNA, or proteins and the majority modulate transcription in *cis* (affecting nearby genes), although they can also modulate in *trans* (targeting distant genes), acting as scaffolds, molecular decoys and guides for epigenetic modifying complexes. Interestingly, lncRNAs can both activate and suppress target genes by a variety of mechanisms and are expressed in a cell-type and stage-specific manner (48, 49). They have been shown to play key roles in autoimmunity, cancer and infection (50–52). A recent comprehensive transcriptomic profiling of T cells demonstrated unique lncRNA signatures for specific T cell phenotypes signifying the relevance of lncRNA to cell and stage specific function (49). Thus, lncRNAs may represent exciting precise therapeutic targets.

## PRC1, PRC2, G9A, and lncRNAs in the Adaptive Immune System

The development of T cells, an integral component of the adaptive immune system, occurs in the thymus where thymocytes mature into distinct T cell lineages defined by either CD4 or CD8 co-receptor expression. CD4+ T cells and CD8+ T cells are known to possess conventional alpha beta (αβ) T cell receptors (TCR), which recognize antigen-derived peptides bound by major histocompatibility complex (MHC) class II or I molecules, respectively. Upon antigen recognition and inflammatory environmental cues, naïve CD4+ T cells differentiate into distinct effector T helper (Th) subsets by expressing lineage-specific transcriptional programs. Th1, Th2, and Th17 cells mediate protective anti-pathogenic responses against bacteria and viruses via the secretion of distinct IFN-γ, IL-4, and IL-17 effector cytokines, respectively (53). Post-infection, Tregs, a regulatory component of the immune system, are recruited to inhibit effector T cell functions and reestablish homeostasis. Tregs can be generated from the thymus (natural Tregs) or induced in the periphery (pTreg) or *in vitro* (iTreg) from naïve CD4+ T cells via a FOXP3-driven transcriptome (54–56). Nonetheless, persistent activation of these effector T cell subsets has been associated with the pathogenesis of autoimmune disorders such as inflammatory bowel disease (IBD), rheumatoid arthritis (RA) and psoriasis (57).

**Figure 1:**
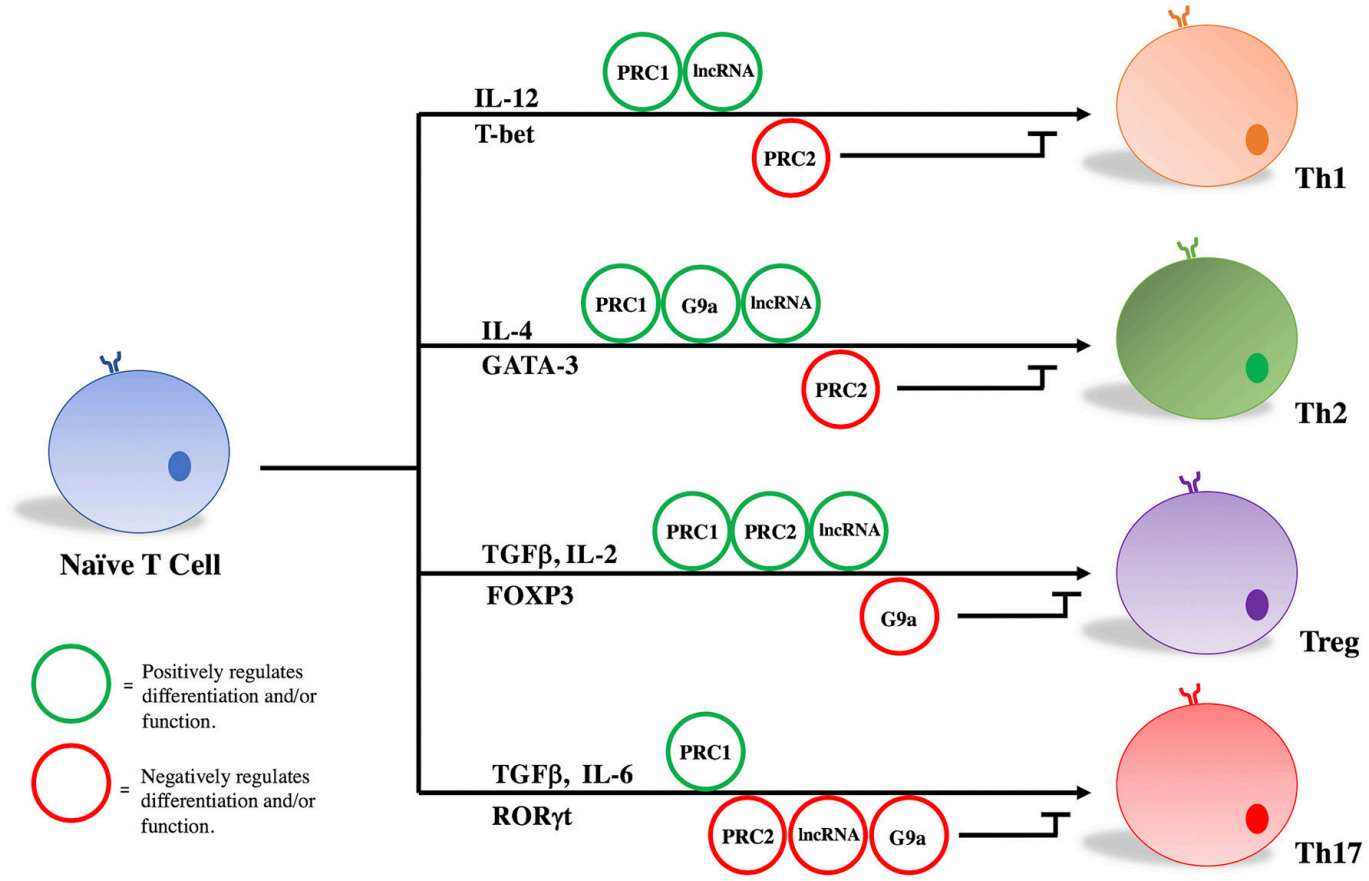
PRC1, PRC2, G9a, and lncRNAs regulate T cell differentiation and function.

PRC1, PRC2, G9a, and a variety of lncRNAs influence T helper cell differentiation and maintenance by epigenetically regulating transcriptional programs associated with different T cell subsets. Given their significant influence in the pathogenicity of diseases as stated above, we focus here on the role of these molecules in the differentiation and maintenance of Th1, Th2, Treg, and Th17 phenotypes (Figure 1, Table 1).

**Table 1:** Roles of PRC1, PRC2, G9a, and annotated lncRNAs in the development and function of Th1, Th2, Treg, and Th17 cells.

	**Th1**	**Th2**	**Treg**	**Th17**
PRC1	Absence of Bmi1 impacts Th1 generation and maintenance (58).	Regulates Th2 differentiation and cytokine expression (59, 60). Overexpression of Bmi-1 increases GATA3 expression and stability (59). Loss of Mel-18 impacts Th2 differentiation *in vivo* (60).	Maintains Treg signature gene expression (61). Inactivation leads to systemic immune mediated disease (61).	Knockdown of Mel-18 leads to decreased expression of *IL17A, IL17F*, and *RORC* (62).
PRC2	Inhibits Th1 differentiation and cytokine production (63, 64). EZH2 deficiency enhances production of Th1 cytokines and increased T-bet expression (63, 64).	Inhibits Th2 differentiation and cytokine production (63, 64). EZH2 deficiency enhances production of Th2 cytokines and increased GATA3 expression (63, 64).	EZH2 is required to promote the FOXP3-mediated gene repression program following TCR stimulation (65). Loss of EZH2 in Tregs *in vivo* leads to multi-organ inflammation and increases susceptibility to experimental models of autoimmunity (66, 65).	EZH2-deficient naïve CD4+ T cells stimulated under Th17 polarizing conditions displayed enhanced production of IL-17 (63).
G9a	No evidence supports a role for G9a in Th1 biology.	Required for Th2-specific cytokine expression (40). Loss of G9a prevents Th2 differentiation and increases IL-17A expression (40, 42).	Absence of G9a in CD4+ T cells is associated with increased FOXP3 expression (42). G9a expression in CD4+ T cells is necessary for development of colitis in mice (42).	Absence of G9a in CD4+ T cells is associated with increased IL-17A expression *in vivo* and *in vitro* (42). Recruited by RelB to silence *IL17A* locus in mouse model of EAE (67).
LncRNA	Linc-MAF-4 promotes Th1 differentiation through silencing of Th2 transcription factor MAF (49, 68). IFNG-AS1 recruits H3-K4-methyltransferase to *Ifng* locus and is upregulated in response to Th1-polarizing cytokines (52, 69, 70, 71).	Th2-LCR-lncRNA recruits WDR5-containing complexes to Th2-specific cytokine loci facilitating their expression (72). LincR-CcR2-5′ AS interacts with GATA-3 to upregulate chemokine genes necessary for Th2 migration (73).	Flicr negatively regulates FOXP3 leading to decreased Treg function (74). Lnc-Smad-3 regulates TGFβ mediated Treg differentiation by interacting with HDAC1 (75). Lnc-EGFR promotes Treg differentiation through interactions with EGFR (51).	Overexpression of LncRNA-1700040D17Rik was associated with decreased expression of RORγt and IL-17 in Th17 cells (76).

### Treg/Th17

Treg and Th17 cells appear to share precursor lineage as demonstrated by *in vitro* study and murine lineage tracing experiments (77, 78). While TGFβ signaling is required for both effector cell types, IL-6 appears principally responsible for ultimate derivation of Th17 cells (79–81). Ultimately, lineage-specific transcription factors (FOXP3 and RORγt) drive the Treg or Th17 transcriptional program, respectively. FOXP3 and RORγt are known to reciprocally regulate one another, and the delicate balance between suppressive Tregs and effector Th17 cells has proven critical for maintaining immune homeostasis (78). Epigenetic modifying complexes, namely PRC2 and G9a, play key roles in orchestrating the Treg and Th17 transcriptional programs, and disruption of these epigenetic networks are characterized by the development of autoimmunity in murine models of human disease and human inflammatory bowel disease (66, 82, 83).

We and others have demonstrated that mice lacking EZH2 in natural FOXP3+ Tregs developed spontaneous multi-organ inflammation and were more susceptible to experimental models of autoimmunity (65, 66). In addition to decreased frequency of EZH2-depleted Tregs observed in certain murine tissues, DuPage et al. showed that EZH2 was required to promote the FOXP3-mediated gene repression program upon TCR activation as a number of FOXP3-bound genes were de-repressed in the absence of EZH2 (65). In support of the failure of EZH2-deleted Tregs to maintain the expression of Treg-specific signature genes, EZH2-deleted Tregs displayed impaired suppression of effector T cells *in vitro* (65, 66). Translating these findings from mice to human relevance, Crohn's disease (CD)-lamina propria CD4+ T cells were transcriptionally different from healthy controls (66). Specifically, normally repressed FOXP3-target genes were upregulated in CD CD4+ T cells and approximately 50% of these differentially expressed genes (DEGs) were EZH2 targets. Moreover, CD4+ T cells displayed a Th1/Th17 effector-like phenotype in contrast to that of healthy controls. Thus, loss of EZH2 function and consequently Treg dysfunction may drive pathophysiological mechanisms of particular autoimmune disorders.

In G9a deficient CD4+ T cells stimulated under Treg or Th17 promoting conditions, a significant increase in FOXP3-expressing and IL-17A-expressing cells is observed. In undifferentiated T cells, G9a normally functions as a mediator of H3K9me2 on loci associated with driving Treg and Th17 phenotypes (42). Loss of G9a-mediated H3K9me2 increases chromatin accessibility to transactivating factors and increases responsiveness to TGFβ (42). Much more work is required to define the molecular underpinnings of G9a's effects on Treg development, but some consistency is emerging regarding Th17 biology. G9a was shown to be recruited by RelB, a non-canonical NF-κB family member, to silence the *IL17A* locus and prevent Th17-mediated autoimmunity in an *in vivo* model of experimental autoimmune encephalomyelitis (EAE) (67). This work is consistent with effects seen in other T cell subsets, namely Th2 cells, in which loss of G9a leads to abnormal IL-17 expression (42). How these effects influence the balance between Treg and Th17 phenotypes is yet to be determined. Thus, G9a may become a viable target for therapeutic intervention of human Th17 mediated diseases.

Three lncRNAs (Flicr, Lnc-Smad-3, and LncEGFR) have been shown to influence Treg function. Flicr is selectively expressed in both human and mouse T regulatory cells and negatively regulates FOXP3 in *cis* leading to decreased Treg function and heightened autoimmunity (74). Mechanistically, Flicr modifies chromatin accessibility in the FOXP3 locus, specifically non-coding sequence 3 (CNS3) and accessible region 5 (AR5), leading to decreased expression of FOXP3. *In vivo*, knockdown of Flicr decreased the incidence of autoimmune diabetes in mice (74).

Lnc-Smad-3 was recently shown to modulate TGFβ-mediated Treg polarization both in human and murine assays (75). Mechanistically, lnc-Smad3 prevents the histone deacetylase HDAC1 to bind to the SMAD3 promoter region, which renders the chromatin compact and inaccessible to Ash1l, an H3K4 methyltransferase that promotes SMAD3 activation and transcription. From a disease relevance standpoint, these results suggest a potential role for this long non-coding RNA in the pathogenesis of autoimmune diseases, such as rheumatoid arthritis (75).

Lnc-EGFR was shown to stimulate Treg differentiation by a forward-feedback loop (51). Mechanistically, lnc-EGFR binds to EGFR using its R1 domain, preventing interaction with c-CBL and ubiquitination. In turn, EGFR activates ERK1/2 and AP-1, which then leads to increased expression of lnc-EGFR and FOXP3, perpetuating increased Treg differentiation. The authors found this to be a critical pathway for hepatocellular carcinoma (51).

LncRNA-1700040D17Rik was found to be deregulated in CD4+ cells derived from a mouse model of autoimmune encephalitis and have been shown to play a role in differentiation of Th17 cells. *In vitro*, overexpression of lncRNA-1700040D1Rik decreased expression of RORγt and IL-17 in Th17 cells, although the precise mechanism is yet to be known (76). These findings suggest a potential role for this long non-coding RNA in the pathogenesis of multiple sclerosis.

### Th1/Th2

Studies investigating the impact of G9a on Th1 biology have shown that the absence of G9a has little effect on Th1 responses *in vitro* nor *in vivo*, however, it is a critical component of the Th2 regulatory machinery (40). Lehnertz et al. demonstrated G9a to be necessary for expression of lineage-specific Th2-associated cytokines such as IL-4, and that loss of G9a in CD4+ T cells prevents Th2 cell differentiation. Mice with targeted CD4+ T cell deletions of G9a were susceptible to helminth infection by *Trichuris muris* due to the inability to express Th2-associated cytokines. Consistent with previous work (42), the absence of G9a in CD4+ T cells also resulted in the upregulation of IL-17A *in vivo*. Interestingly, whereas repression of IL-17A appears to be associated with G9a methyltransferase activity (42), Th2 gene regulation by G9a is independent of enzymatic activity, and thought to be related to G9a functioning as a scaffolding protein (40, 41).

The role of PRC1 in regulating T cell lineage fate decisions is best illustrated by the influence it has on the Th1/Th2 axis of development. Both Bmi1 (PCGF 4) and Mel-18 (PCGF 2) have been shown to physically interact with GATA3, a lineage specific transcription factor for Th2 differentiation, in a Ring finger dependent manner (59, 60). Mel-18 has been shown to regulate *GATA3* transcription, and knockout of *mel-18* severely impacts Th2 differentiation *in vivo* (60). Bmi-1 regulates Th2 cell differentiation by acting as an inhibitor of GATA3 degradation and regulator of its stability. Bmi-1 overexpression in itself leads to an increase in GATA3 expression and an increase in Th2 cell differentiation under a Th2 specific cytokine milieu. Comparatively little data exist regarding the role of PRC1 in Th1 cell development/function; however adoptive transfer of CD4+ T cells from *Bmi1*^−/−^ mice into nude mice showed impaired generation and maintenance of memory Th1 cells through Bmi1-mediated repression of *Noxa*, a pro-apoptotic gene (58).

The role of EZH2 in modulating effector T function was recently illuminated by Yang et al. who showed that EZH2-deficient naïve CD4+ T cells stimulated under Th1, Th2 or Th17 polarizing conditions displayed enhanced production of IFN-γ, IL-13 or IL-17 cytokines, respectively (63). Moreover, Tumes et al. also showed that EZH2 deficiency in naïve CD4+ T cells led to the upregulation of Th1 and Th2-associated cytokines with concomitant increase in lineage-specific transcription factors T-bet and Gata3, respectively (64). However, *in vivo* studies have revealed that EZH2 plays a dichotomous role in the differentiation and senescence of CD4+ T cells (63). For example, in an *in vivo* model of *Listeria monocytogenes* infection known to induce a Th1 response, CD4+ T-specific EZH2 deleted mice displayed impaired clearance of infection due to decreased survival of memory Th1 cells (84). Additionally, OVA-specific EZH2-deficient Th2 cells were pathogenic in a mouse model of allergic asthma due to an accumulated and exaggerated immune response from memory Th2 cells (64). Taken together, EZH2 inhibits effector cytokine production in naïve CD4+ T cells, and loss of EZH2 enhances differentiation to effector Th cells as well as effector Th cell plasticity. Based on evidence from *in vivo* studies in mice in the context of EZH2 deletion in T cells, effector Th cell dysfunction is consistent across all disease models, evidently through impaired clearance of pathogens or aggravated autoimmunity (potentiated tissue destruction). Additionally, H3K27me3-independent functions of EZH2 have been reported in T cells expressing conventional αβ-TCRs (17, 18). Vasanthakumar et al. demonstrated that EZH2 prevents NKT cell expansion through methylation, ubiquitination and subsequent degradation of the transcription factor promyelocytic leukemia zinc finger (PLZF) (17). *In vivo* studies have demonstrated that an increase in the frequency of NKT cells in the thymus and spleen occurs as a result of CD4+ T-specific EZH2 deletion, which may contribute to the perturbed immunity seen in murine studies previously mentioned (63, 64, 84).

Two lncRNAs, MAF-4, and IFNG-AS1 (also called NeST or Tmevpg1), have been shown to influence Th1 biology by recruiting different epigenetic modifying complexes. Linc-MAF-4 is selectively expressed in Th1 cells and promotes Th1 differentiation through epigenetic silencing of the Th2 transcription factor MAF. Downregulation of linc-MAF-4 in human CD4+ cells skewed differentiation toward a Th2 phenotype. Mechanistically, linc-MAF-4 promotes a *cis* chromatin looping conformation, leading to the recruitment of chromatin remodelers EZH2 and LSD1 that place repressive H3K27me3 marks on the promoter region of MAF-4 silencing its expression (49). Recently, linc-MAF-4 was shown to be involved in the pathogenesis of multiple sclerosis by promoting Th1 cell differentiation (68). Thus, far, linc-MAF-4 has not been studied *in vivo*.

IFNG-AS1 is expressed in CD4+ Th1, CD8+, and natural killer cells (52, 69). It is upregulated in CD4+ cells in response to Th1-differentiating cytokine stimuli and plays a critical role in transcription of *Ifng*. This has been demonstrated both *in vitro* and *in vivo*. Mechanistically, it has been shown to recruit the H3K4-methyltranferase complex to the *Ifng* locus, leading to placement of activating marks at the promoter region. It has been associated with the pathogenesis of Hashimoto's thyroiditis (70), ulcerative colitis (71), and the immune response to viral infections *in vivo* (52).

Two lncRNAs, Th2-LCR-lncRNA and lincR-CcR2-5′AS, have been shown to influence the development and function of Th2 cells. Th2-LCR-lncRNA is selectively expressed in human Th2 cells and is transcribed in the RAD50 locus and epigenetically regulates expression of IL-4, IL-5 and IL-13 (72). Mechanistically, Th2-LCR-lncRNA recruits WDR5-containing complexes to targeted cytokine loci, enhancing transcription. Knockdown of human Th2-LCR-lncRNA *in vitro* causes major loss of expression of IL-4, IL-5 and IL-13 in Th2 cells through loss of H3K4me3 activating marks (72). Unfortunately, Th2-LCR-lncRNA is not conserved in mice, complicating *in vivo* studies.

LincR-CcR2-5′AS is selectively expressed in mouse Th2 cells and upregulates *CCR1, CCR2, CCR3* and *CCR5* chemokine genes in a GATA3-dependent fashion (73). Interestingly, knockdown of this lincRNA not only affected neighboring genes *CCR2* and *CCR3*, but also affected nearly 1,200 genes some of which were located in distant loci, suggesting it can act in both *cis* and *trans*. Although the precise mechanism is yet to be fully understood, *in vitro* knock down of lincR-CcR2-5′AS did not result in chromatin accessibility or modification of H3K4me3, suggesting that it does not act through recruitment of histone-modifying enzymes or chromatin structure modifications.

## Future Perspectives: Epigenetic Modulation of T cells in Clinical Practice

Epigenetic mechanisms of disease are in theory inducible and reversible through environmental manipulation, however, some epigenetic features have been shown to be maintained after cellular division as a result of self-enforcing feedback mechanisms (85). The heritable, yet reversible nature of epigenetic therapy makes this a promising option for treatment. Persistence of epigenetic maintenance of engineered modifications has been shown to be stable up to 40 days post modification induction *in vivo* (86). Most epigenetic drugs currently in use inhibit DNA methyltransferase and histone deacetylase activity, and have been shown to reverse immune suppression and thus sensitize the host immune system in combination with anti-cancer therapies. Several anti-cancer mechanisms have been reported, such as enhancing antigen processing and presenting machinery pathways, inhibiting immune checkpoints, and enhancing chemokine production. For patients, there are three treatment options available: therapies reported to affect DNA methylation, inhibitors of histone post-translational modifications, and compounds interfering with non-coding RNA regulation (87). Repurposing drugs and screening for new compounds that display converse effects to treatment autoimmune disease is an exciting new option for autoimmune illnesses.

Distinct DNA methylation profiles have been demonstrated in CD8+ and CD4+ T cells isolated from patients experiencing autoimmune diseases (88–90). Epigenetic based therapeutics currently being employed for the clinic for non-inflammatory conditions, such as arrhythmias (procainamide), hypertension (hydralazine), and neoplasia (5-azacytidine), have been shown to induce auto-reactive pathology (7, 8). However, the 5-azacytidine derivative 5-aza-2'deoxycytidine, which is also a DNA methyltransferase inhibitor used in hematological malignancies, has been shown to have a positive outcome when administered in animal models of diabetes (91), colitis (92), multiple sclerosis (93), and graft-versus-host-disease (GvHD) (94). We need a better understanding of the implications of DNA methylation, the pharmokinetics of available compounds, and synergistic effects of combination therapy with immunomodulatory drugs already in practice for autoimmune diseases to allow us to develop and implement novel therapies. As of now, we are lacking a therapeutic arsenal to target global hypomethylation, which is most often associated with lymphocytes recovered from patients experiencing some of the most common autoimmune diseases.

The ubiquitous expression of EZH2 and the opposing role it plays in different cell-types makes EZH2 a delicate therapeutic target. Recent identification of PRC2- and H3K27me3-independent EZH2 functions in oncogenesis indicates that a complete suppression of all oncogenic functions of EZH2 is required to combat cancer. Anti-EZH2 therapy inhibits methylation at key repression/silencing associated histone marks, and these compounds have emerged as a promising therapy for cancer treatment, especially for B cell non-Hodgkin's lymphoma. However, we have observed that systemic anti-EZH2 therapy leads to mucosal hypersensitivity in mice. One complicating factor is that EZH2 is also utilized by PRC1 in the nucleus, therefore more study needs to be undertaken to dissect the specific roles these complexes play in inflammation before on can determine whether histone methyltransferase inhibitors can be co-opted for anti-inflammatory therapy. Of note, cytosolic forms of PRC2 have been shown in murine models to be necessary for TCR-mediated activation of signaling pathways that drive T cell proliferation and autoimmunity. Thus, pharmacologic targeting of cytosolic PRC2 may represent a more precise therapeutic approach to suppressing autoimmunity caused by excessive T cell activation (19, 20).

From a translational standpoint, several studies have demonstrated that long non-coding RNAs can be used as biomarkers in malignancy and autoimmune diseases (95–97). Potential lncRNA-targeted therapeutic approaches include silencing by antisense base pairing (e.g., targeting lncUBE3ATS, which silences paternal UBE3A in Angelman's syndrome) or by targeting molecules that are necessary for lncRNA transcription, such as transcription factors (98, 99). The cell type specific expression of lncRNAs makes them excellent targets for therapeutic intervention, as off-target effects are minimized. One option being pursued in cancer therapies is to directly target HOTAIR; a primarily *trans*-acting long-non coding RNA that promotes gene silencing through recruitment of PRC2 and LSD1 complexes, resulting in trimethylation of H3K27 and demethylation of H3K4, respectively (100–102). Knocking down HOTAIR provides compelling evidence for therapeutic targeting in cancer. Arresting glioblastoma multiform cell migration and invasion through this approach is a case in point (103). To overcome the limitation of genetic targeting, peptide nucleic acids have been developed which disrupt complex function. This approach has had positive results in inhibiting NF-κB activity in addition to decreasing ovarian and breast cancer properties such as reduced tumor formation and survival (104). The potential for this approach in inflammatory diseases is still to be determined.

Precision medicine has brought about the advent of using CRISPR/Cas9 to target this gene editing tool to target epigenetic modifying enzymes to precise locus specific locations on the genome instead of the DNA endonucleases the technology originally utilized (105). This technique can be exploited to recruit enzymes that impact the methylation of the DNA, enzymes that post-translationally modify the histones, and proteins which interfere with non-coding RNA regulation. Further, it has been recently reported CRISPR/Cas9 technology can be rapidly delivered via a non-viral delivery technique capable of integrating large DNA sequences (106). These new developments will allow us flexible and precise epigenetic manipulation toward creating therapeutically epi-engineered primary human immune cells without the off-target effects associated with systemic epigenetic therapies.

## Author Contributions

WF contributed conception and design of the manuscript. JG, MB, GR, AB, MG, MS, and OS wrote sections of the manuscript. JG wrote the first draft of the manuscript. All authors provided critical revision and final approval of the manuscript.

### Conflict of Interest Statement

The authors declare that the research was conducted in the absence of any commercial or financial relationships that could be construed as a potential conflict of interest.

## Footnotes

^1^*GENCODE, v27 Release*. Available online at: https://www.gencodegenes.org/human/release_27.html

^2^*Long Non-coding RNA Database v2.,0 (lncRNAdb)*. Available online at: http://www.lncrnadb.org/
